# BARRIERS AND FACILITATORS OF THE INNOVATION PROCESS IN LONG-TERM CARE HOMES DURING COVID-19

**DOI:** 10.1093/geroni/igae098.2163

**Published:** 2024-12-31

**Authors:** Suzanne Santos, Yanjun Duan, Gilciney Andrade Rabello, Sumaya Bhatti, Charlene Chu, Michael Lepore, Franziska Zuniga, Ruth Melo

**Affiliations:** University of São Paulo, São Paulo, Sao Paulo, Brazil; University of Toronto, Toronto, Ontario, Canada; University of São Paulo, São Paulo, Sao Paulo, Brazil; University of Toronto, Toronto, Ontario, Canada; University of Toronto, Toronto, Ontario, Canada; University of Massachusetts Amherst, Amherst, Massachusetts, United States; University of Basel, Basel, Basel-Stadt, Switzerland; University of São Paulo, São Paulo, Sao Paulo, Brazil

## Abstract

The innovation process is complex and multidimensional regardless of the context in which it is applied. However, innovation sustainability can be even more challenging when applied in the Long-Term Care (LTC) sector. So, understanding the barriers and facilitators associated with innovation adoption can be essential to innovation sustainability in LTC homes. In the qualitative studies incorporated within the scoping review (n =18), an in-depth analysis was conducted to discern barriers and facilitators related to innovation in LTC homes during COVID-19. Barriers to innovation identified were related to residents (e.g., safety, privacy, and appropriateness), staff (e.g., workload and tight routine), LTC homes (e.g., infrastructure and material/human resources), and innovation itself (e.g., cost, technical and connectivity problems). Concerns about the inclusiveness of the residents, especially those with some health-related issues (e.g., dementia and macular degeneration) were also pointed out. Conversely, innovations are likelier to be adopted if they prove to be useful and convenient, align with the needs of both LTC homes and residents, and exhibit user-friendly features. From an organizational standpoint, facilitating innovation during COVID-19 involves preparing the LTC home, providing necessary equipment, and offering training and support to staff. Familiar members/caregivers also point as a positive factor the ability of innovation in relief of care burden. Codesign approaches, involving different stakeholders, might be essential to identifying barriers and facilitators and, therefore, contribute to the sustainability of the innovations inside LTC homes.

